# Exploring the Impact of Urolithins A and B on Muscle Health: A Transcriptomic Analysis in Human Myotubes

**DOI:** 10.1002/jcsm.70385

**Published:** 2026-09-23

**Authors:** Yves Henrotin, Antoine Florin, Christelle Sanchez, Prescilia Centonze, Tiago Pinto Coelho, Sophie Bekisz, Jean‐Emile Dubuc, Olivier Galand, Cécile Lambert

**Affiliations:** ^1^ musculoSKeletal Innovative research Lab (mSKIL), Center for Interdisciplinary Research on Medicines (CIRM), University of Liège, Institute of Pathology, CHU Sart‐Tilman Liège Belgium; ^2^ Physical Therapy and Rehabilitation Department Princess Paola Hospital Marche‐en‐Famenne Belgium; ^3^ Groupe Interdisciplinaire de Génoprotéomique Appliquée (GIGA), Metabolism and Cardiovascular Sciences University of Liege Liège Belgium; ^4^ Biomechanics Research Unit, GIGA Molecular & Computational Biology University of Liège Liège Belgium; ^5^ Orthopaedic Department University Clinics St Luc Brussels Belgium; ^6^ Orthopaedic Department Bois de l'Abbaye Hospital Seraing Belgium

**Keywords:** inflammation, musculoskeletal biology, myotubes, RNA sequencing, urolithins

## Abstract

**Background:**

Urolithin A (UA) and urolithin B (UB) are gut microbiota‐derived metabolites of ellagitannins reported to influence mitochondrial function, inflammation and muscle metabolism. Their comparative transcriptomic effects in human skeletal muscle cells remain undefined. We characterized UA‐ and UB‐induced molecular responses in primary human myotubes.

**Methods:**

Primary CD56^+^ satellite cells were isolated from *vastus lateralis* muscle of 9 donors (6 men, 3 women; age 55–96 years; mean 74.1 ± 13.8 years) and differentiated into myotubes. Cells were treated 24 h with UA or UB (5 μM). RNA sequencing generated ~20 million paired‐end reads per sample. Differential expression analysis was performed using DESeq^2^ (design = ~Patient + Treatment). Differentially expressed genes were defined as adjusted *p* value (FDR) < 0.01 and |Log2FoldChange| > 0.32. Pathway enrichment was assessed using Ingenuity Pathway Analysis. Selected targets were validated by RT‐qPCR and ELISA in 4 donors from the RNA‐seq cohort using UA and UB at 1, 5 and 10 μM.

**Results:**

UA and UB significantly modulated 1918 and 339 genes, respectively (FDR < 0.01; |log2FoldChange| > 0.32), demonstrating distinct transcriptomic reprogramming in human myotubes. Pathway analysis showed that UA predominantly affected oxidative phosphorylation, mitochondrial dysfunction, inositol phosphate metabolism and glycosylation pathways (N‐linked glycosylation *z* score 2.11), whereas UB activated cholesterol biosynthesis (*z* score 2.45), mevalonate pathway (*z* score 2.00), adipogenesis (*z* score 1.41) and inhibited eicosanoid signalling (*z* score −2.71). At 5 μM (RNA‐seq), UA increased NOTCH1 (+73%), MYMX (+70%), PANX1 (+50%) and MSTN (+64%), and decreased FGF9 (−75%), ICAM5 (−52%) and MRLN (−33%), whereas UB decreased IGFN1 (−75%), TGFBI (−60%) and STC2 (−35%) and increased TGM2 (+59%). UA increased LIF (+80%) and decreased PTGS1 (−41%) and IL17B (−49%), whereas UB decreased PTGS1 (−43%) and increased IL17B (+45%). RT‐qPCR validation confirmed UA‐induced increases in NOTCH1 (5 μM, *p* = 0.0471), MYMX (10 μM, *p* = 0.0363), and PANX1 (5 μM, *p* = 0.0012; 10 μM, *p* = 0.0065), dose‐dependent reductions in FGF9 (*r* ^2^ = 0.9389) and ICAM5 (*r* ^2^ = 0.8804), and opposite regulation of IL17B (UA *r* ^2^ = 0.8309; UB 10 μM, *p* = 0.0498) and PTGS1. Both UA and UB reduced TGFBI protein levels dose‐dependently (UA *r* ^2^ = 0.8582; UB *r* ^2^ = 0.7415).

**Conclusions:**

UA and UB induce quantitatively and qualitatively distinct transcriptomic programmes in human myotubes. UA preferentially modulates mitochondrial and inflammatory pathways, whereas UB primarily affects lipid metabolism and muscle‐related processes. These findings provide mechanistic insight into urolithin‐mediated regulation of human muscle cell biology able Stro.

## 1 Introduction

Skeletal muscle ageing is a health problem, associated with a progressive decline in muscle mass, strength and motor control. The result is a clinical condition known as *sarcopenia*. Sarcopenia is now defined as a loss of muscle mass combined with alterations in physical function and muscle structure and performance [1, 2]. This pathology is thus recognized not only as an ageing problem but one associated with a range of long‐term conditions impacting both patient mobility and mortality. Today, the World Health Organization recognizes sarcopenia as a disease and includes it in the International Classification of Disease [3].

In terms of prevalence, sarcopenia increases with age up to 29% in older persons in the community healthcare setting and ranges from 11% to 50% in persons aged 80 years and above [2, 4]. Among risk factors, we can cite nutritional aspects (such as low protein intake, malabsorption, anorexia, …), causes associated with inactivity (immobility, sedentary life, …), diseases (bone and joint diseases, metabolic disorders, endocrine disorders, …) and finally, iatrogenic (hospital admission or drug‐related) [5]. No specific drug has been approved for the treatment of sarcopenia; only nonpharmacological approaches have been considered, such as physical activity, caloric restrictions and nutritional supplements, including amino acid supplementation [6, 7]. At the same time, pharmacological interventions such as selective androgen receptor modulators or myostatin inhibitors were also evaluated. However, these focused on increasing muscle mass rather than muscle strength to circumvent this age‐related decline in muscle function [8, 9, 10, 11].

In recent years, evidence has suggested that urolithins have health benefits due to their anti‐inflammatory, anti‐cancer, neuroprotective and cardioprotective effects. These gut microbiota metabolites of ellagitannins (ETs) and ellagic acid (EA), two polyphenols found in fruits (such as pomegranate and berries), nuts and wood‐aged wine, were discovered almost 20 years ago [12]. These metabolites are beneficial in delaying many age‐related diseases, including cardiovascular diseases, chronic metabolic disorders and cancer [13]. ETs are dietary oligomeric polyphenols that release EA upon hydrolysis, but their bioavailability ranges from negligible (ETs) to very low (EA) [14]. Both reach the colon, where the gut microbiota metabolizes them into urolithins through lactone‐ring cleavage, decarboxylation and dihydroxylation [14]. Urolithin bioconversion varies between individuals, resulting in three gut microbiota‐associated Uro metabotypes: metabotype A (only Uro‐A), metabotype B (Uro‐B, IsoUro‐A and Uro‐A), and metabotype 0 (no urolithin production) [15]. To date, 13 urolithins and their conjugated metabolites have been detected in various fluids and tissues.

In terms of muscle health, urolithin A (UA) has been shown to promote *mitophagy*, *mitochondrial function* and *improved muscle function* in both preclinical and clinical studies [16, 17]. Urolithin B (UB) has recently emerged as a ‘newly identified regulator’ of skeletal muscle mass, promoting myotubes differentiation and hypertrophy [18]. Additionally, UB has been found to exert testosterone‐like effects, positively regulating muscle protein balance [19].

Given these findings, we aimed to evaluate the effects of UA and UB, the primary metabolites of ellagitannins, on the human myotube transcriptome by comparing gene expression profiles in human primary muscle cells treated with either UA or UB.

## 2 Methods

### 2.1 Ethical Statement

Skeletal muscle biopsies were obtained post‐mortem from the *vastus lateralis* of nine donors (six men and three women; mean age 74.1 ± 13.8 years, range 55–96 years) via an anonymized tissue bank. Donors were not prospectively recruited following strict exclusion criteria like myopathies or other neuromuscular conditions, and available clinical information was limited to age, sex, post‐mortem delay and cause of death. None of the donors had a documented history of primary myopathies or neuromuscular diseases. The protocol was approved by the ethical committee of the University of Liège (Ref. 2018/237). All procedures followed the ethical standards of the responsible committee on human experimentation (institutional and national) and the Helsinki Declaration of 1975, revised in 2000.

### 2.2 Human Muscle‐Derived Cell Culture

Briefly, muscle biopsies were dissociated mechanically into small fragments of around 1–2 mm^3^. These fragments were subjected to digestion with 4.8 mg/mL dispase II (Sigma‐Aldrich, Bornem, Belgium), 1 mg/mL collagenase *clostridium histolyticum* type 1A (Sigma‐Aldrich) and 2.5‐mM CaCl_2_2H_2_O in Dulbecco's modified Eagle's medium (DMEM; Lonza, Verviers, Belgium) supplemented with 10‐mM HEPES, 100 units/mL penicillin and 100 mg/mL streptomycin (Lonza) at 37°C for 1 h. The cell suspension was passed through a 70‐mm filter to remove undigested tissue. The filtered cell suspensions were collected by 800 g centrifugation, and cells were then sorted by immunomagnetic sorting using CD56 Microbeads, a transmembrane glycoprotein expressed on the surface of satellite cells (Miltenyi Biotech, Leiden, The Netherlands). Satellite cells were cultured in DMEM supplemented with 10‐mM HEPES, 100 units/mL penicillin, 100 mg/mL streptomycin, 2‐mM glutamine, 10% fetal calf serum (BioWest, Nuaillé, France) and 2% Ultroser G (PALL Life Sciences, Cergy‐Saint‐Christophe, France) renewed three times per week. Cells were then differentiated during 48 h into myotubes with DMEM supplemented with 10‐mM HEPES, 100 units/mL penicillin, 100 mg/mL streptomycin, 2‐mM glutamine and 0.5% Ultroser G. Myotube differentiation was confirmed by measuring the expression of myogenic markers, such as myosin by RT‐qPCR and through myogenesis highlighted by immunofluorescence analysis (Figures S1 and S2). Finally, myotubes were subsequently treated during 24 h with UA and UB at 1–10 μM. UA and UB were dissolved in DMSO, and control cells received the same final concentration of DMSO (0.05%, *v*/*v*) as vehicle control. For transcriptomic analyses, differentiated human myotubes were treated with UA or UB at a single concentration of 5 μM for 24 h. This concentration (5 μM) was selected to balance biological activity with physiological relevance. Multiple independent studies have shown that urolithins exert robust effects on mitochondrial function, mitophagy and muscle‐related pathways at low micromolar concentrations, typically within the 1‐ to 10‐μM range [16, 20]. Importantly, pharmacokinetic studies indicate that UA can reach low micromolar plasma concentrations in humans following supplementation, making 5 μM compatible with achievable exposure rather than a supraphysiological condition [21]. This concentration therefore represents a biologically active, nonextreme condition suitable for unbiased transcriptomic profiling and direct comparison of UA and UB effects in myotubes.

### 2.3 DNA Quantification

DNA content of cell culture was measured according to a fluorometric method using Hoechst [22].

### 2.4 RNA Extraction

Total RNA was extracted using a RNeasy mini kit (Qiagen Inc., Netherlands) according to the manufacturer's instructions. The extract yield was determined spectrophotometrically by measuring the optical density at 260 nm. The purity and quality of extracted RNA were further evaluated using the RNA Nano 6000 Bioanalyzer Agilent (Santa Clara, United States) according to the manufacturer's instructions. High‐quality RNAs with an RNA quality indicator score (RIN) of > 8 were used.

### 2.5 RNA Sequencing and Differential Gene Expression Analysis

This analysis used 100 ng of RNA from each culture condition. Libraries were prepared with the Illumina Truseq stranded mRNA sample prep kit according to the manufacturer's instructions. Sequencing was performed on Novaseq (Illumina), paired end reads (150–10–10–150), NovaSeq S4 V1.5 300‐cycle XP workflow, generating around 20 M reads per sample. After filtering out reads mapping to rRNA, tRNA, mitochondrial RNA and other contaminants (e.g., adapters, etc.) using bowtie2, reads were aligned onto the human reference genome (GRCh38 and Ensembl 106 annotation) and quantified with STAR to give the Counts file. Quality control of sequencing reads was assessed with FASTQC, and quality control was assessed after mapping with Picard tools. Differential expression analysis was made in R (version 4.4.0, https://www.R‐project.org/) using the DESeq2 package (1.44.0)^S1^, biomaRt package (version 2.60.0)^S2^ and R code design = ~Patient + Treatment. Analysis was performed with treatment as a contrast: each treated vs. control and the combination vs. each extract separately. Volcano plots and Venn diagrams were generated using ggplot2 (version 3.5.1) and nVennR (version 0.2.3)^S3^. DEseq2 data were analysed with the use of QIAGEN IPA version 111 725 566 (QIAGEN, https://digitalinsights.qiagen.com/IPA). A false Discovery Rate (FDR) of 0.01 was used to assess the statistical significance. For more details, please refer to supporting the information.

Validation experiments (RT‐qPCR and ELISA) were performed on a subset of four donors selected based on technical criteria, including sufficient CD56^+^ myogenic cell yield and robust differentiation capacity, due to limited availability of biological material from primary human muscle cultures.

### 2.6 Real‐Time Reverse Transcriptase

Total RNA was extracted using RNeasy mini kit (Qiagen) according to the manufacturer's instructions and was reverse transcribed with Sensiscript Reverse Transcriptase (Qiagen) according to the manufacturer's instructions. The cDNAs were quantified by quantitative real‐time Polymerase Chain Reaction (qPCR) using the Rotor Gene instrument (Qiagen) and the SYBR Premix Ex Taq kit (Takara, Verviers, Belgium). The level of gene expression was determined by interpolation with a standard curve. The ribosomal s18 was amplified as an internal control to standardize mRNA levels. The primer sequences used were shown in Table S1.

### 2.7 ELISA for TGFBI

The protein amount of TGFBI was measured from the supernatant by a specific enzyme‐amplified sensitivity immunoassay (Human beta IG‐H3 DuoSet ELISA DY2935). Protein content was normalized to DNA content.

### 2.8 Statistical Analysis

The DESeq2 Bioconductor package was used for normalization, principal component analysis (PCA) and differential gene expression for the transcriptome analysis. DESeq2 differential gene analysis was based on the hypothesis that most genes were not differentially expressed^S1^. The method was based on the negative binomial distribution model. Scaling factors were calculated for each run within the DESeq2 package and with the estimate SizeFactorsForMatrix function. After dividing gene counts by each scaling factor, DESeq2 values were calculated as the total of rescaled gene counts of all runs. The amplitude of changes was represented either in the log2foldchange format (classical representation from DESeq2, where ‘0’ means ‘no change’, ‘1’ means ‘2‐fold induction’ and ‘−1’ means ‘2‐fold decrease’) or in fold change (where ‘1’ means ‘no change’, ‘2’ means ‘2‐fold induction’ and ‘0.5’ means ‘2‐fold decrease’). Along with the standard *p* value, an adjusted *p* value (*p*_adj_) was calculated. The adjustment methods included the Bonferroni correction (‘Bonferroni’) in which the *p* values were multiplied by the number of comparisons. Less conservative corrections were also included by Holm (‘holm’) (Holm 1979), Hochberg (‘hochberg’)^S4^, Hommel (‘hommel’)^S5^, Benjamini & Hochberg (‘BH’ or its alias ‘fdr’)^S6^, and Benjamini & Yekutieli (‘BY’)^S7^, respectively. The ‘BH’ (aka ‘FDR’) and ‘BY’ methods of Benjamini, Hochberg and Yekutieli control the false discovery rate, that is, the expected proportion of false discoveries among the rejected hypotheses.

Results for the protein analyses and gene expression were statistically analysed using GraphPad Prism 6.0. The mean was calculated for the triplicate of each cell culture. These values were first analysed in GraphPad Prism 6.0 to check their normality (D'Agostino & Pearson omnibus test). Then, paired one‐way ANOVA was used to compare each treatment to the control, with multiple‐comparison correction (Holm–Sidak's) or Friedman test for repeated measures with Dunn's post‐test, to calculate the statistical significance.

## 3 Results

### 3.1 Global RNA‐Seq Analysis

**FIGURE 1:**
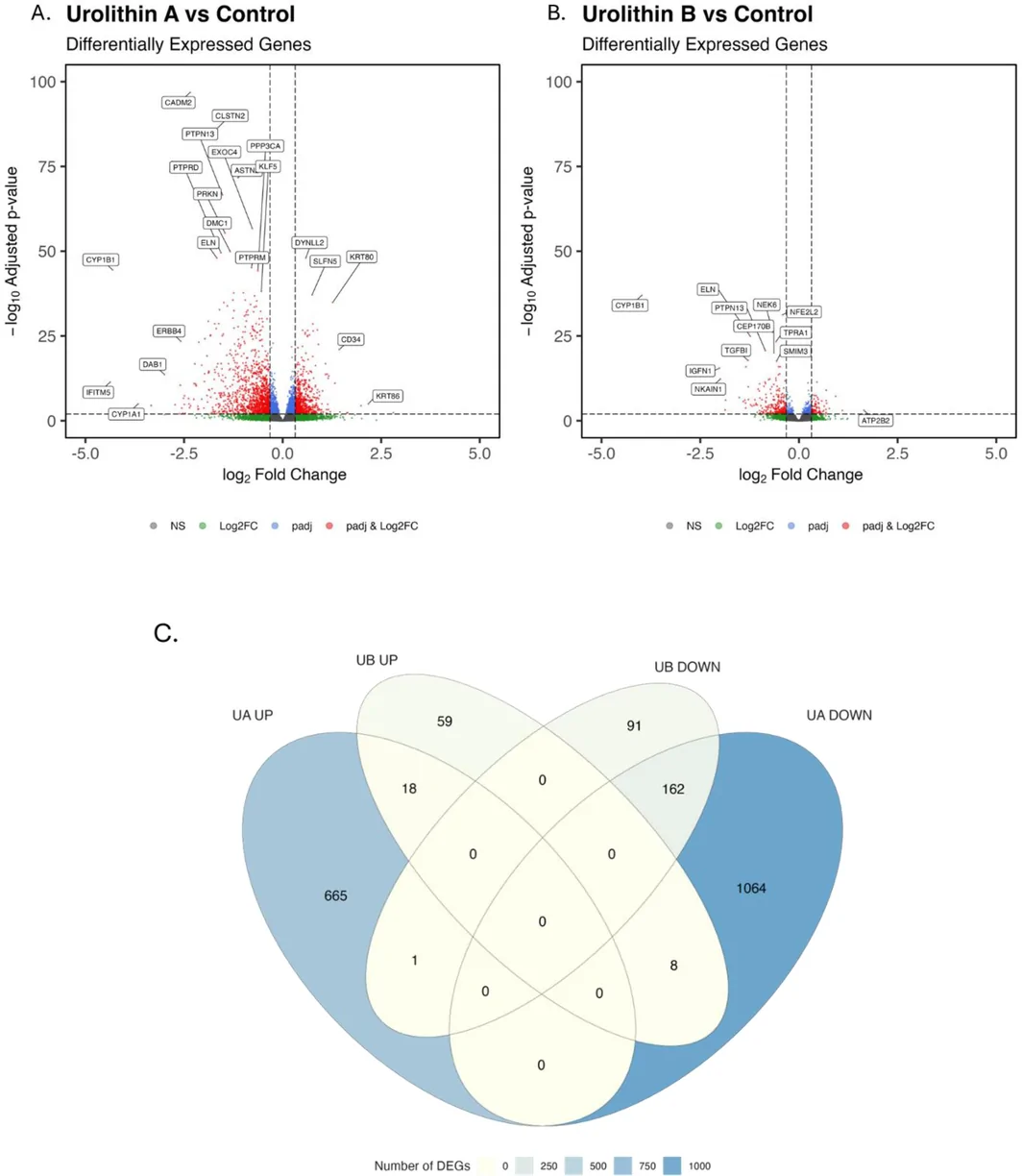
Volcano plots (A,B) and Ven diagram (C) of UA (5 μM) and UB (5 μM) after 24‐h treatment on human myotubes. Threshold *p*_adj_ < 0.01 and Log2FoldChange > |0.32|, *n* = 9. DEGs: differentially expressed genes.

After 24 h of treatment, UA and UB at a concentration of 5 μM significantly modified the expression of 1918 and 339 genes, respectively (Figure 1A,B). A Log2FoldChange threshold of 0.32 (~1.25‐fold) was applied to identify differentially expressed genes (DEGs), reflecting the expectation of modest but biologically meaningful transcriptional changes in primary human samples. Statistical significance was assessed using adjusted *p* values (*p*_adj_ < 0.01). As illustrated in the Venn diagram (Figure 1C), 162 genes were down‐regulated by both UA and UB, while 18 genes were commonly up‐regulated (Table S2). Nine genes exhibited opposite expression patterns between UA and UB. Of these, eight were up‐regulated by UB and down‐regulated by UA, while only one was up‐regulated by UA and down‐regulated by UB (Table 1).

**TABLE 1:** List of genes presented opposite expression patterns between UA and UB. UA: urolithin A (5 μM) and UB: urolithin B (5 μM). Threshold *p*_adj_ < 0.01 and Log2FoldChange > |0.32|.

UB up and UA down		UA up and UB down	
Gene symbol	Protein	Gene symbol	Protein
EPB41L1	Erythrocyte membrane protein band 4.1 like 1	NQO1	NAD(P)H quinone dehydrogenase
JPH1	Junctophilin‐1		
GOT1	Glutamic‐oxaloacetic transaminase 1		
IL‐17B	Interleukin 17B		
ESYT1	Extended synaptotagmin 1		
BEAN1	Brain expressed associated with NEDD4 1		
PCSK9	Proprotein convertase subtilisin/kexin type 9		
SLC04C1	Solute carrier organic anion trasnporter family member 4c1		

### 3.2 Effects of UA on Myotubes Transcriptome

**FIGURE 2:**
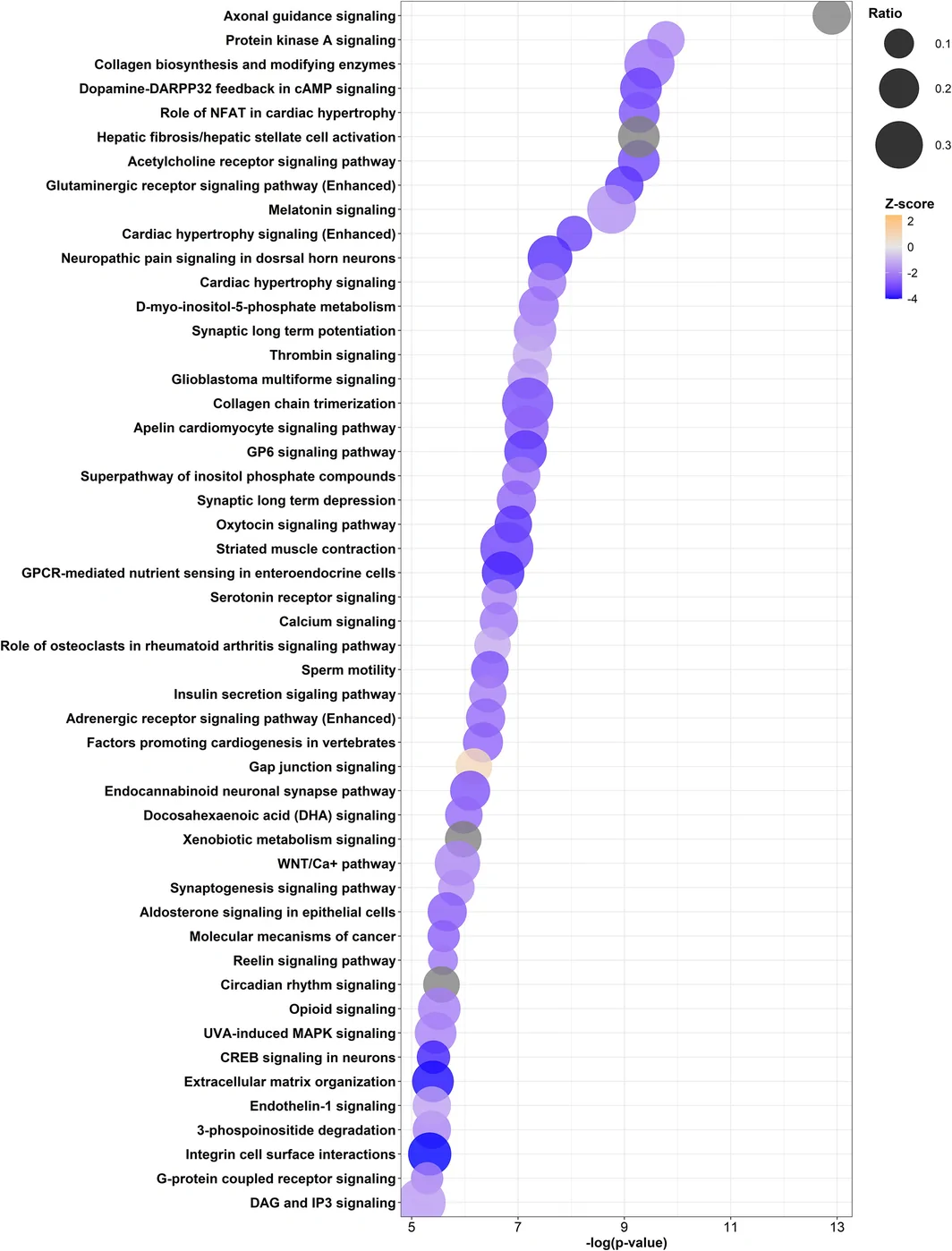
Top 50 canonical pathways identified by Ingenuity Pathway Analysis (IPA) following urolithin A (UA) treatment. Pathways are ranked by decreasing significance (−log(*p* value), *x*‐axis). Bubble size represents the ratio of differentially expressed genes in each pathway, while bubble colour indicates the IPA activation *z* score, predicting pathway activation (positive values) or inhibition (negative values).

The list of differentially expressed genes (DEGs) was subjected to core analysis using the Ingenuity Pathway Analysis (IPA). The top 50 canonical signalling pathways were identified by sorting them from largest to smallest based on the −log (*p* value) and were presented in Figure 2. Several pathways involved in muscle physiology have been identified: *D‐myo‐inositol‐5‐phosphate Metabolism*, *Calcium Signalling*, *O‐linked glycosylation* and *the Asparagine N‐linked glycosylation pathway*. Except for the Asparagine N‐linked glycosylation pathway (*z* score 2.11), all the pathways mentioned above exhibited a negative *z* score, suggesting that most of these pathways are inhibited by UA.

**FIGURE 3:**
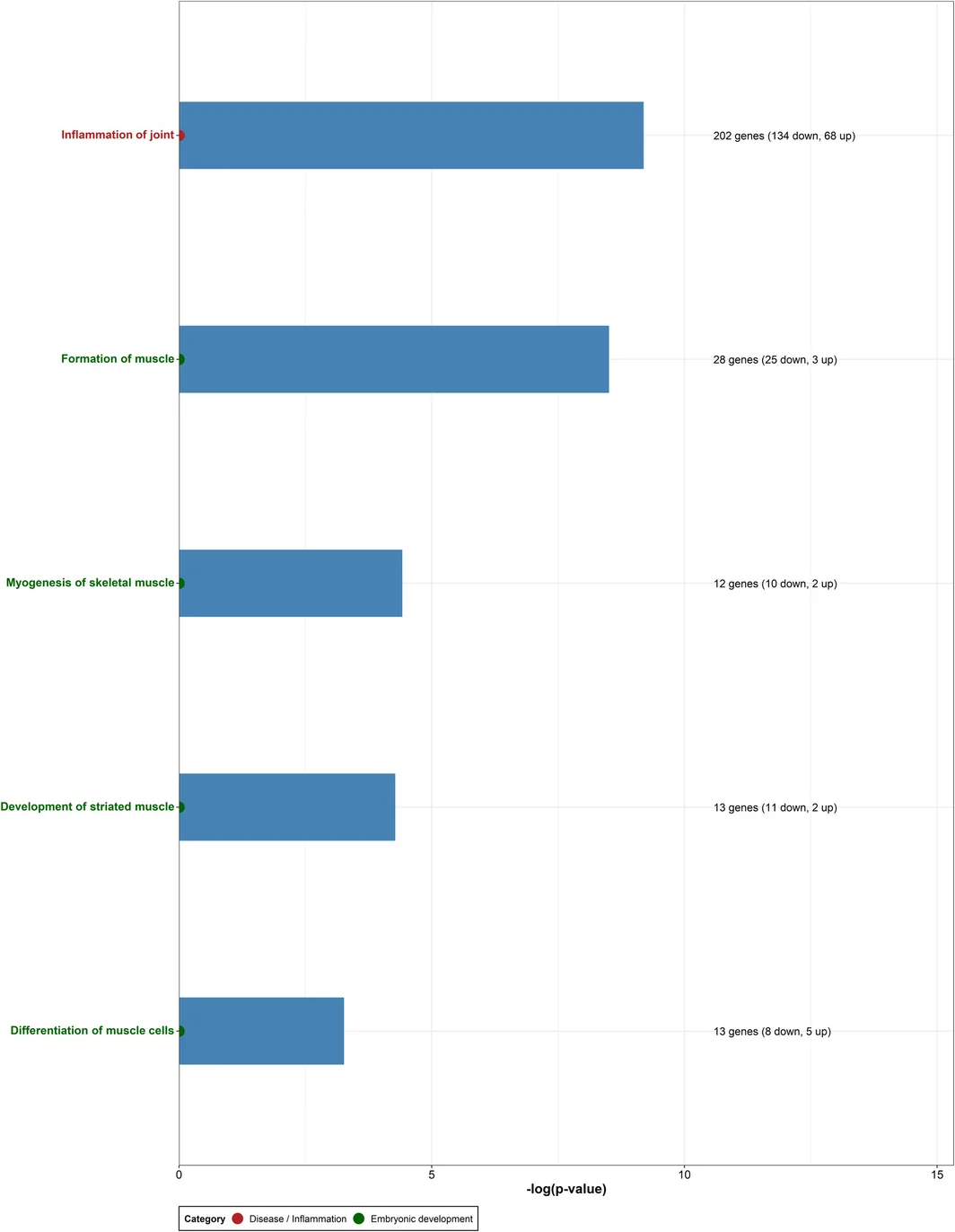
Key biological functions and associated diseases in response to urolithin A (UA, 5 μM). Biological functions and disease categories are ranked according to their significance (−log(*p* value), *x*‐axis). The total number of differentially expressed genes associated with each function is indicated, together with the numbers of down‐regulated and up‐regulated genes. Coloured dots denote the corresponding functional category.

To understand the biological implications of our findings, we analysed gene expression based on their biological functions and involvement in diseases. This analysis revealed significant enrichment in two main categories: ‘Embryonic development, organ development, organismal development, skeletal and muscular system development and function, tissue development’; and ‘Connective tissue disorders, Inflammatory disease, Inflammatory Response, Organismal injury and abnormalities, Skeletal and muscular disorders’ (Figure 3 and Table S3). The first category highlighted the substantial impact of UA on various aspects of skeletal muscle development. Notably, we observed significant effects on skeletal muscle myogenesis (*p* value 3.82E−05; with 2 genes up‐regulated and 10 genes down‐regulated), striated muscle development (*p* value 5.28E−05; with 2 genes up‐regulated and 11 genes down‐regulated), muscle cell differentiation (*p* value 5.42E−04; with 5 genes up‐regulated and 8 genes down‐regulated) and muscle formation (*p* value 3.11E−09; with 3 genes up‐regulated and 25 genes down‐regulated). In contrast, the second category indicated UA's effects on joint inflammation, with a significant *p* value of 6.39E−10, revealing 67 genes up‐regulated and 136 genes down‐regulated.

### 3.3 Effect of UB on Myotubes Transcriptome

**FIGURE 4:**
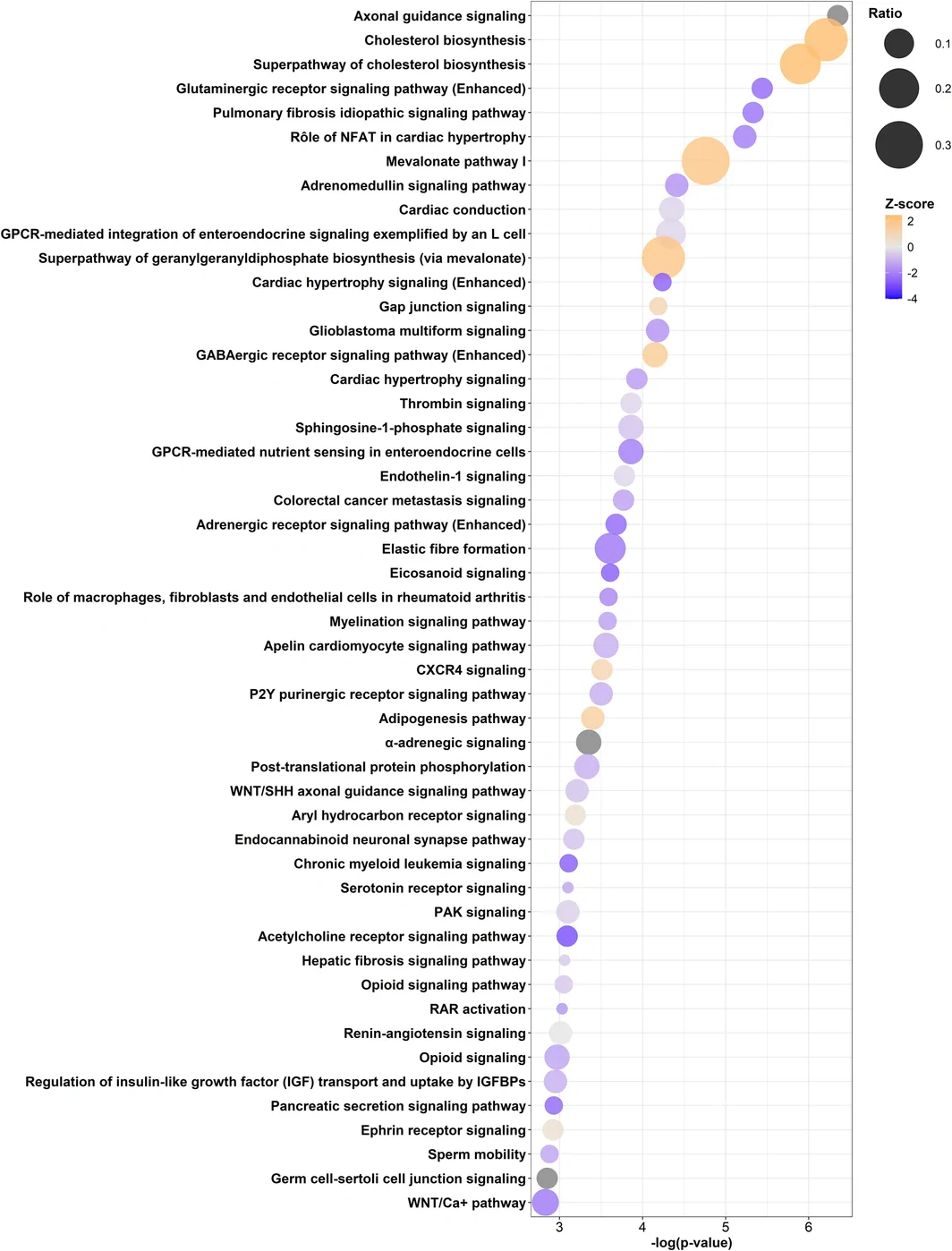
Top 50 canonical pathways identified by Ingenuity Pathway Analysis (IPA) in response to urolithin B (UB). Canonical pathways are ranked according to their significance (−log(*p* value), *x*‐axis). Bubble size represents the ratio of differentially expressed genes from our dataset relative to the total number of genes assigned to each canonical pathway. Bubble colour indicates the IPA activation *z* score, with positive values predicting pathway activation and negative values predicting pathway inhibition.

Similar to UA, the top 50 canonical signalling pathways were identified and are presented in Figure 4. ‘*Cholesterol biosynthesis*’ and ‘Superpathway of cholesterol biosynthesis’ pathways emerged from this analysis with high *z* score (2.45) indicating a strong activation of these pathways by UB. To a lesser extend ‘Mevalonate pathway I’ and ‘Superpathway of geranylgeranyldiphosphate biosynthesis’ (*z* score 2.00) and ‘*Adipogenesis pathway*’ (*z* score 1.41) were also activated by UB. In contrast, UB down‐regulated ‘the eicosanoid signaling pathway’ (*z* score −2.71), indicating a potential reduction in inflammatory responses and lipid mediator synthesis within the myotubes butup‐regulated ‘the CXCR4 signaling pathway’ (*z* score 1.13), crucial for muscle regeneration via satellite cells.

**FIGURE 5:**
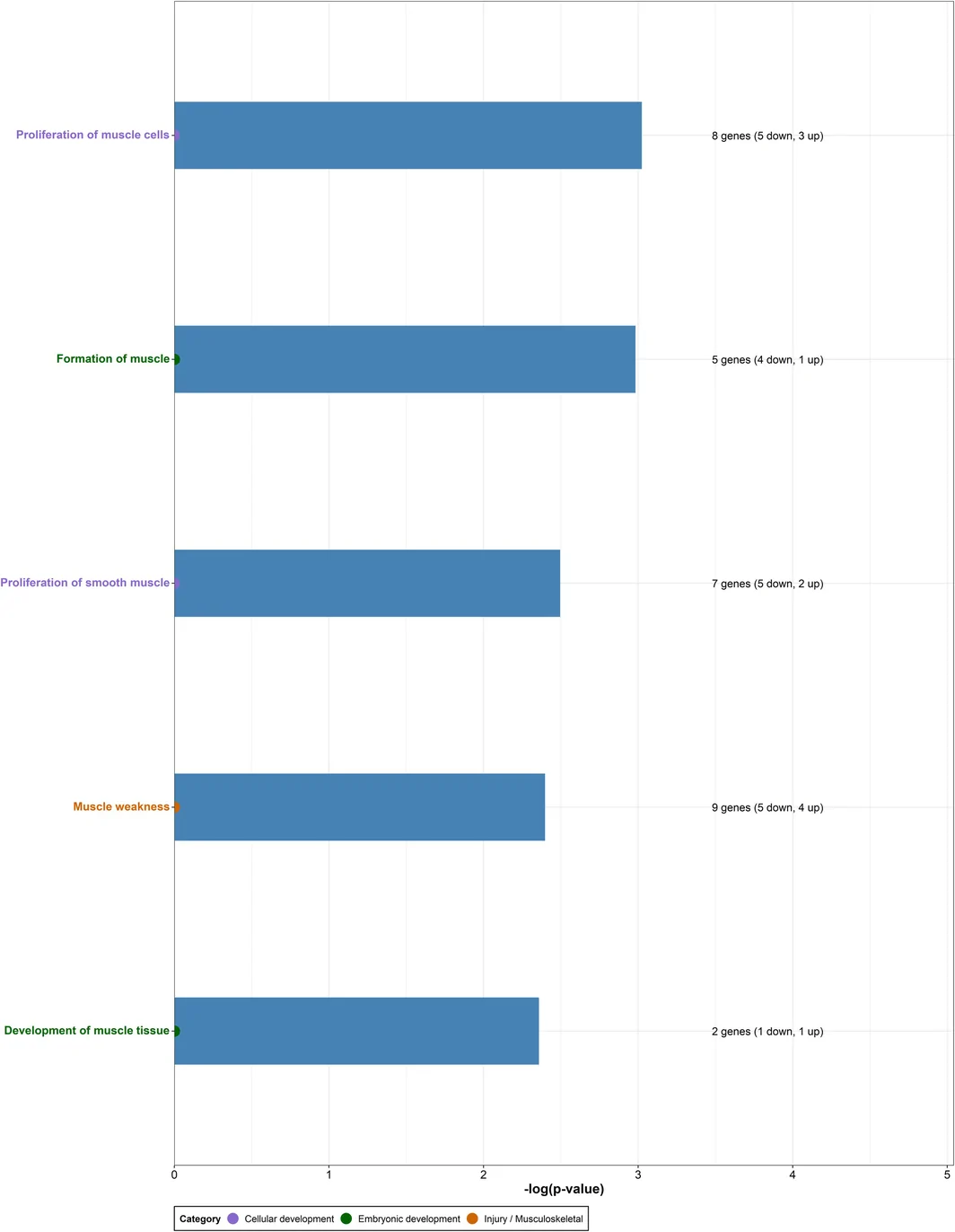
Key biological functions and associated diseases identified by Ingenuity Pathway Analysis (IPA) in response to urolithin B (UB, 5 μM). Biological functions and disease categories are ranked according to their significance (−log(*p* value), *x*‐axis). The total number of differentially expressed genes associated with each function is indicated, together with the numbers of down‐regulated and up‐regulated genes. Coloured dots denote the corresponding functional category.

The ‘Diseases and Functions’ analysis following UB treatment demonstrated substantial enrichment across several critical categories, including ‘Cellular development, Cellular growth and proliferation, Organ development, Skeletal and muscular system development and function, and Tissue development’; ‘Embryonic development, Organ development, Organismal development, Skeletal and muscular system development and function, and Tissue development’; and ‘Organismal injury and abnormalities, Skeletal and muscular disorders’ (Figure 5 and Table S4). Specifically, 8 genes (with 3 genes up‐regulated and 5 genes down‐regulated) were regulated by UB in the proliferation of muscle cells (*p* value 9.46E−04) category, 7 genes (with 2 genes up‐regulated and 5 genes down‐regulated) in the proliferation of smooth muscle cells (*p* value 3.19E−03) category, 5 genes (1 up‐regulated and 4 genes down‐regulated) in muscle formation category (*p* value 1.04E−03), 2 genes (1 genes up‐regulated and 1 genes down‐regulated) in development of muscle tissue category (*p* value 4.38E−03) and 9 genes (with 4 genes up‐regulated and 5 genes down‐regulated; *p* value 4.00E−03) in the muscle weakness category.

### 3.4 Distinct and Common Pathways by UA and UB

Pathways such as Oxidative phosphorylation and Mitochondrial dysfunction were predominantly influenced by UA, while *myogenesis*, *muscle cell proliferation* and *apelin signalling pathways* were mainly regulated by UB. Some pathways were commonly modulated by both UA and UB but with varying degrees of activation or inhibition, such as the *Calcium Signalling Pathway*, *GPCR (G Protein‐Coupled Receptor) Signalling Pathway*, *mTOR Signalling Pathway*, *Oxidative Stress Response* or *Integrin Signalling Pathway* (Figure S3).

### 3.5 Effect of UA and UB on Genes Involved in the Skeletal and Muscular Systems

The most up‐regulated genes of the skeletal and muscular system by UA (5 μM) were NOTCH1 (+73%), MYMX (+70%), PANX1 (+50%), MSTN (+64%) and the most down‐regulated were FGF9 (−75%), MRLN (−33%) and ICAM5 (−52%) whereas UB (5 μM) decreased IGFN1 (−75%), TGFBI (−60%), STC2 (−35%) and increased TGM2 (+59%).

**FIGURE 6:**
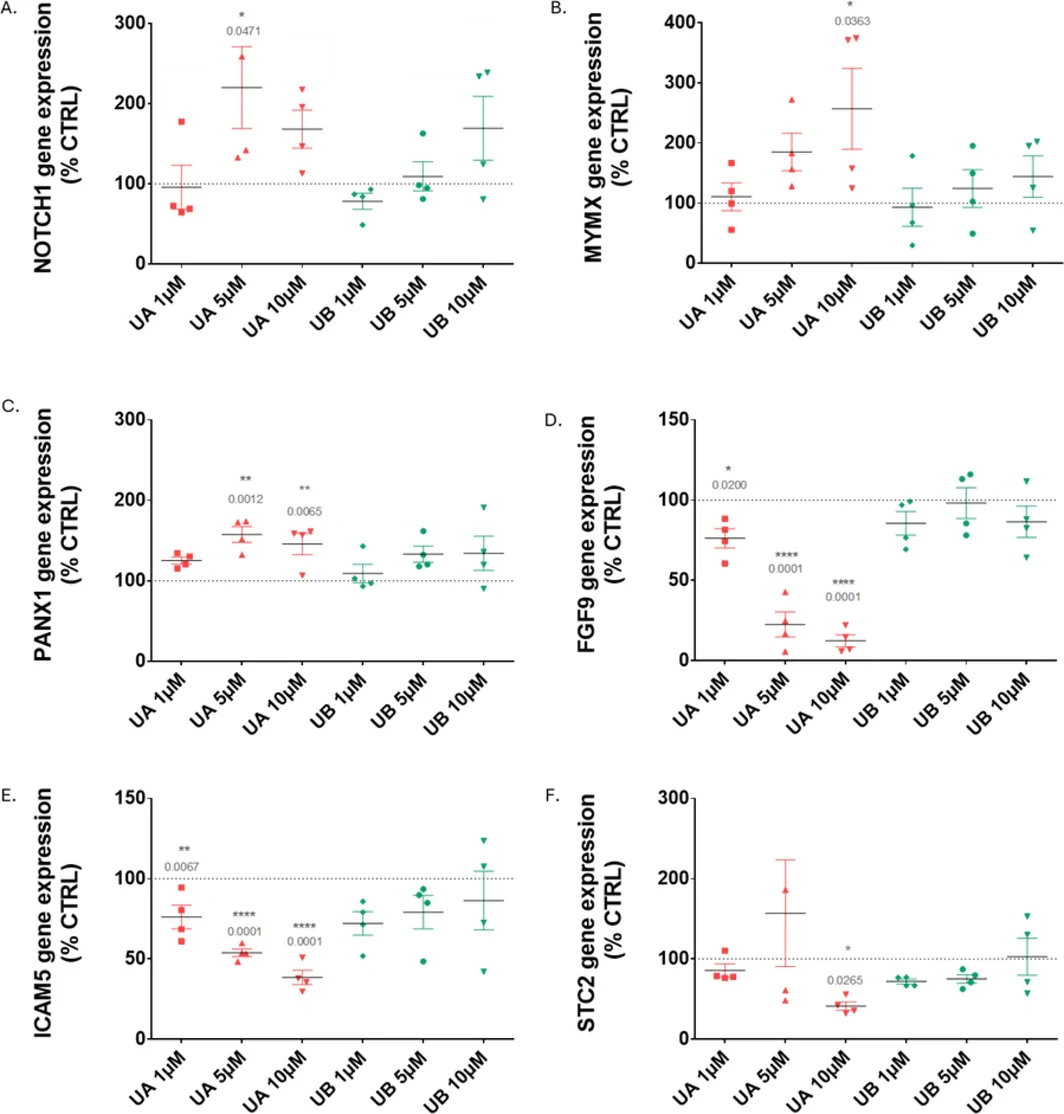
Modulation of target regulators of skeletal muscle. NOTCH1 (A), MYMX (B), PANX1 (C), FGF9 (D), ICAM5 (E) and STC2 (F). Myotubes were treated 24 h with UA (1, 5 and 10 μM) or UB (1, 5 and 10 μM). Total RNA was isolated in cellular extract and NOTCH1, MYMX, PANX1, FGF9, ICAM5 and STC2 were analysed by real‐time PCR technology. Results are expressed as per cent control (Ctl) and represented by mean with 95% CI (*n* = 4). *p < 0.05, **p < 0.01, ***p < 0.001 and ****p < 0.0001.

**FIGURE 7:**
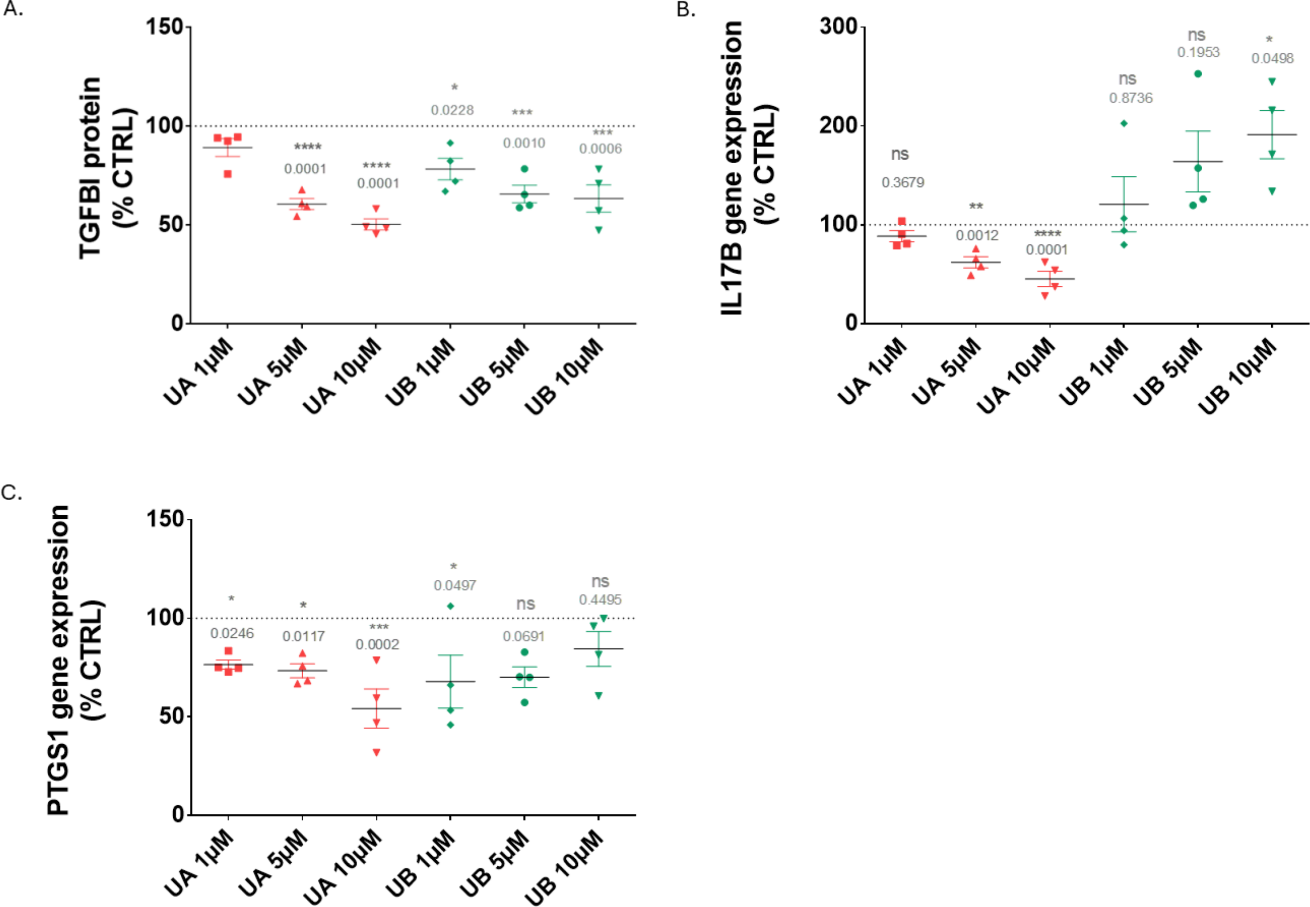
Modulation of TGFBI protein and inflammatory gene expression. Myotubes were treated 24 h with UA (1, 5 and 10 μM) or UB (1, 5 and 10 μM). TGFBI protein levels (A), a regulator associated with skeletal muscle remodelling, were determined by ELISA and normalized to DNA content. IL‐17B (B) and PTGS1 (C) gene expression levels were analysed by real‐time PCR technology as markers involved in the inflammatory process. Total RNA was isolated in cellular extract. Results are expressed as per cent control (Ctl) and represented by mean with 95% CI (*n* = 4). *p < 0.05, **p < 0.01, ***p < 0.001 and ****p < 0.0001.

We then evaluated the dose–response effects of urolithins on the expression of several genes identified as highly regulated in the transcriptomic analysis. UA increased the expression of NOTCH1, a key regulator of muscle cell fate and differentiation, at the concentration of 5 μM (*p* = 0.0471), consistent with the RNA‐sequencing results (Figure 6A). UA also increased the expression of MYMX, a muscle‐specific membrane protein with the ability to regulate the myogenic fusion process, at the concentration of 10 μM (*p* = 0.0363) and PANX1, a member of Pannexins family and recently identified as a novel regulator of skeletal muscle myoblast proliferation and differentiation, at the concentration of 5 (*p* = 0.0012) and 10 μM (*p* = 0.0065) (Figure 6B,C) but dose‐dependently decreased FGF‐9 (*r* ^2^ = 0.9389) and ICAM5 (*r* ^2^ = 0.8804) (Figure 6D,E). UB decreased the expression of STC2 at the concentrations of 1 and 5 μM; however, this effect did not reach statistical significance (Figure 6F). UB was not effective on these genes at any dose tested (Figure 6A–E). Finally, we evaluated the effect of urolithins on TGFBI production. Both UA and UB significantly reduced TGFBI production in muscle cells in a dose‐dependent manner (*r* ^2^ = 0.8582 and *r* ^2^ = 0.7415, respectively) (Figure 7A).

### 3.6 Urolithins Influence Myoblast Differentiation Dynamics

Using Nanolive CX‐A technology, we monitored the real‐time effects of UA and UB (5 μM) on myoblast differentiation. Fusion‐like events were observed as early as 6 h after treatment with UA and UB, while control cells showed minimal fusion at this time point (Figure S4). Manual quantification of nuclei was performed at different time points. Nuclei were classified as isolated (mononucleated myoblasts) or located in close proximity within continuous cellular structures, consistent with multinucleated myotubes. The proportion of isolated nuclei relative to total nuclei per field was determined. After 24 h, UA‐ and UB‐treated conditions showed a reduction in isolated nuclei compared with control, consistent with enhanced incorporation of myoblasts into multinucleated structures (Figures S4 and S5). These findings are presented as supportive evidence within the context of the broader molecular data.

### 3.7 Modulation of Genes Involved in the Inflammatory Process

Transcriptomic analyses using DESeq2 revealed that urolithins modulate several genes involved in the inflammatory process. In particular, LIF expression was increased by 80% in response to UA, whereas PTGS1 expression was decreased by 41% and 43% following UA and UB treatment, respectively. In addition, UA and UB exerted opposite effects on IL‐17B expression, with a 49% decrease induced by UA, and a 45% increased induced by UB, as identified in the RNA‐sequencing dataset (Table S5).

To further validate these transcriptomic changes, dose–response RT‐qPCR analyses were performed on selected inflammation‐related genes (IL‐17B and PTGS1) in myotubes derived from four donors. As shown in Figure 7B, IL‐17B was dose‐dependently decreased by UA (*r* ^2^ = 0.8309) while UB (*p* = 0.0498 at UB 10 μM; *r* ^2^ = 04141) dose‐dependently increased this factor. Similar data was obtained for the PTGS1 gene. UA dose‐dependently decreased PTGS1 while UB increased it (Figure 7C). Together, these RT‐qPCR results confirm the RNA‐sequencing data, supporting a differential and compound‐specific modulation of inflammation‐related genes by UA and UB.

## 4 Discussion

Skeletal muscle is crucial for mobility and quality of life, particularly in ageing populations, where sarcopenia, a condition characterized by progressive loss of muscle mass and function, has emerged as a major clinical challenge. Despite advances in understanding the molecular and cellular mechanisms underlying muscle degeneration, effective pharmacological interventions for sarcopenia remain limited, with targeted therapies still in early development stages [23]. The rising prevalence of sarcopenia due to ageing underscores the urgency of identifying interventions that can preserve muscle health. In this context, dietary polyphenols, such as ellagitannin‐derived metabolites UA and UB, have growing attention for their potential therapeutic benefits.

In this study, we aimed to elucidate the molecular mechanisms by which UA and UB impact muscle health at the transcriptional level. Using a transcriptomic approach, we provide a comprehensive analysis of gene expression profiles in human myotubes treated with UA and UB. To our knowledge, this represents the first direct comparison of the transcriptomic effects of UA and UB in human muscle cells. While UA has been extensively studied able Stro, there is still limited knowledge regarding the impact of UB.

Regarding muscle health, UA has been shown to promote muscle protein synthesis and muscle growth, primarily by improving mitochondrial function and regulating autophagy [24]. In contrast, UB has been studied mainly in cardiac muscle tissue [25]. UB has been found to protect against apoptosis, inflammation and neural remodelling in myocardial tissue while inhibiting the expression of gap junction channels, critical elements in arrhythmia. Additionally, UB reduces the occurrence of myocardial arrhythmias following hypoxia via the regulation of the Akt/mTOR pathway and the nuclear translocation of NF‐κB, suggesting its potential in treating ischemic heart disease. Despite this focus on the cardiac muscle, the effects of UB on skeletal muscle remain largely unexplored, highlighting the need for further investigation. Only one study has examined the effects of UB on skeletal muscle, both able Stro and able Svo. This research revealed that UB prevented muscle atrophy and promoted hypertrophy in mice after denervation by enhancing protein synthesis and reducing protein degradation, mainly through the ubiquitin‐proteasome pathway [18]. able Stro, UB similarly promoted the growth and differentiation of C2C12 myotubes by boosting protein synthesis and inhibiting the same degradation pathway.

Our findings provide novel insights into the distinct molecular pathways influenced by these metabolites and suggest their potential as therapeutic agents in muscle‐wasting conditions such as sarcopenia. These results contribute to a deeper understanding of how specific gut‐derived metabolites modulate muscle physiology and underscore the therapeutic potential of targeting the gut‐muscle axis in age‐related muscle decline. One of the most striking findings of our study is the differential regulation of a significant number of genes by UA and UB, with UA modulating the expression of 1918 genes and UB affecting 339 genes. Among these, 180 genes were commonly regulated by both UA and UB, with 18 genes up‐regulated and 162 down‐regulated. This overlap suggests shared pathways through which both urolithins may benefit muscle cells, possibly through their well‐documented antioxidant and anti‐inflammatory properties [26]. However, beyond this overlap, we identified nine genes with opposing expression patterns between UA and UB, indicating that these metabolites may also have distinct and, in some cases, contrasting effects on muscle cells. Eight genes (EPB41L1, JPH1, GOT1, IL17B, ESYT1, BEAN1, PCSK9, SLC04C1) were increased by UB but decreased by UA whereas a single gene, NQO1, was up‐regulated by UA and down‐regulated by UB. Among these discordantly regulated genes, IL‐17B emerged as particularly noteworthy. Although the role of IL‐17B in skeletal muscle physiology remains incompletely defined, recent work in dystrophinopathies has identified IL‐17B as a novel biomarker of human muscle regeneration, with IL‐17B expression enriched in regenerating myofibres and included in a core regeneration‐associated transcriptional signature alongside developmental myosin genes [27]. In that context, IL‐17B correlates with histological indices of necrosis and regeneration rather than with quiescent muscle homeostasis, suggesting that IL‐17B reflects an active tissue remodelling state. The opposite regulation of IL‐17B by UA and UB in our model therefore likely reflects differential tuning of muscle‐intrinsic inflammatory and regenerative transcriptional programmes, rather than a simple pro‐versus anti‐inflammatory dichotomy. Down‐regulation of IL‐17B by UA may be compatible with attenuation of chronic, low‐grade inflammatory signalling, which implicated in the pathogenesis of sarcopenia and other muscle disorders [28]. Conversely, up‐regulation of IL‐17B by UB may reflect engagement of transcriptional pathways associated with an active remodelling or repair‐like state, in which a controlled inflammatory component is required. At this stage, our data support IL‐17B primarily as a sensitive marker of the inflammatory/regenerative state of human myotubes, rather than a fully characterized effector of myotube physiology, and dedicated functional studies will be required to define its direct autocrine or paracrine roles in skeletal muscle. Another essential gene with opposing regulation is NQO1, a gene coding for an enzyme contributing to cellular defence mechanisms against oxidative stress by catalysing the reduction of quinones to hydroquinones, thereby preventing the formation of reactive oxygen species (ROS) [29]. UA significantly up‐regulated NQO1 expression, consistent with its known role in enhancing mitophagy and mitochondrial function. By contributing to the cellular antioxidant defence system, NQO1 helps ensure muscle tissues can sustain their tasks effectively and recover from exercise‐induced damage. In contrast, the down‐regulation of NQO1 by UB raises questions about how muscle cells balance oxidative stress and anabolic processes, two closely linked mechanisms, when exposed to UB.

While both compounds are derived from ellagitannins, their divergent impact on gene expression suggests that they may engage distinct molecular pathways, possibly due to differences in their metabolic processing or receptor interactions. Pathway enrichment analysis provided further insights into the distinct roles of UA and UB. We have deciphered the mechanisms of action of UA and UB and discovered that they modulate different cellular functions, suggesting that the net result of elegantin supplementation could be influenced by the concentration of these two metabolites in the muscle. The core analysis of UA‐treated myotubes highlighted several enriched canonical pathways, including *D‐myo‐inositol‐5‐phosphate Metabolism*, the *O‐linked glycosylation* and the *Asparagine N‐linked glycosylation pathway*. The inositol phosphate system, notably through molecules such as inositol‐1,4,5‐trisphosphate (IP3), plays a crucial role in cellular signalling, metabolic processes and energy regulation, with a specific focus on mitochondrial function [30] Recent literature supports that UA promotes mitophagy and enhances mitochondrial function, improving muscle health [31]. Therefore, if UA moderately inhibits the D‐myo‐inositol‐5‐phosphate pathway, it may trigger a compensatory response, boosting mitophagy and mitochondrial biogenesis, which are crucial for maintaining cellular energy balance. This will ultimately improve muscle health by enhancing energy efficiency and protecting against stress or inflammation. We also observed a significant enrichment of glycosylation‐related pathways with UA, with inhibition of O‐linked glycosylation and activation of the Asparagine N‐linked glycosylation pathway. It is now clear that protein glycosylation is a crucial post‐translational modification involved in maintaining normal muscle structure and function [32] and previous studies have shown that various glycosylation alterations are closely linked to muscle diseases. N‐glycosylation plays a critical role in protein folding, stability, localization and function, while O‐glycosylation is key in modulating protein activity, interactions and intracellular signalling. Thus, the activation of N‐glycosylation by UA could enhance correct protein folding and the stability of membrane plasma proteins, crucial for maintaining cellular integrity and myotube signalling. Conversely, by inhibiting O‐glycosylation, UA could favourably modulate protein–protein interactions and limit certain aberrant post‐translational modifications, thus contributing to cell protection against stress or inflammation. Additionally, UA's effect on muscle development and differentiation is highlighted by the enriched terms ‘Skeletal and Muscular System Development and Function’ and ‘inflammation of the joint’. This suggests a dual role of UA in promoting muscle growth and modulating inflammatory responses, which could be beneficial in mitigating age‐related muscle degeneration and associated inflammatory conditions. According to the IPA, we confirmed in four different patients the effect of UA on several target regulators of skeletal muscle such as MYMX, PANX1 and TGFBI.

UB treatment resulted in a down‐regulation of the *eicosanoid signalling pathway*, implying a potential reduction in inflammatory responses in myotubes. This anti‐inflammatory effect could be advantageous in reducing chronic inflammation associated with sarcopenia [33]. Interestingly, data analysis also highlights the possible beneficial effects of UB on pathways related to cholesterol biosynthesis and lipid metabolism, which could be advantageous for muscle health in an acute, controlled context. Among lipids, cholesterol is a vital component of cell membranes, and it is now well‐established that it plays a crucial role in cellular repair and proliferation during the recovery from muscle injuries [34]. In addition, cholesterol is also a critical component of both the inner and outer mitochondrial membranes, where its lipid composition is known to significantly impact mitochondrial function. Additionally, activating the adipogenesis pathway suggests that UB may also promote lipid storage within muscle tissues. Recent experimental research has highlighted fibro‐adipogenic progenitors (FAPs), also known as muscle‐resident mesenchymal progenitors, as key regulators of muscle homeostasis and integrity, providing trophic and extracellular matrix support to myogenic cells during repair [35]. However, dysregulated or persistent activation of FAPs is also implicated in fibrofatty infiltration and myosteatosis, conditions associated with reduced muscle quality and functional decline [36]. Our data reflect short‐term transcriptional responses in differentiated myotubes and do not allow conclusions about long‐term structural lipid accumulation. We therefore interpret UB‐induced modulation and cholesterol and adipogenesis‐related pathways as a potentially adaptive response that may support membrane turnover, mitochondrial homeostasis and controlled lipid handling in this acute model, while acknowledging that similar pathways, if chronically activated in muscle, are linked to detrimental myosteatosis. This dual aspect of lipid metabolism should be taken into account when considering the potential impact of UB on muscle integrity and function. The up‐regulation of the ‘CXCR4 signaling pathway’ further highlights UB's role in muscle regeneration. Recently, Shams et al. demonstrated the critical role of CXCR4 signalling in satellite cell activation, proliferation and self‐renewal, suggesting that UB might support muscle regeneration and recovery, which are essential aspects of skeletal muscle injury [37].

This study offers promising insights for developing therapeutic strategies against muscle degeneration. UA, with its potent effects on mitochondrial function and inflammation, could be particularly beneficial for older adults or those with chronic conditions leading to muscle atrophy. Its ability to enhance mitophagy and reduce oxidative stress makes it a strong candidate for addressing mitochondrial dysfunction linked to age‐related diseases like sarcopenia. In contrast, UB may be more effective for *muscle regeneration* and *repair*, especially when anabolic processes need enhancement. Its role in activating lipid metabolism and stimulating muscle regeneration pathways suggests the potential for aiding recovery after injuries or surgeries. The distinct yet complementary functions of UA and UB provide a unique opportunity to explore combination therapies targeting both the degenerative and regenerative aspects of muscle health.

Overall, the present study was designed as a mechanistic and exploratory investigation aimed at identifying transcriptional pathways and molecular signatures modulated by UA and UB in human myotubes. In this context, the use of a global transcriptomic approach, complemented by focused phenotypic and molecular validation of selected targets, was intended to provide an unbiased molecular framework rather than an exhaustive functional characterization of all deregulated pathways. This strategy allows the identification of candidate processes and regulatory nodes that can serve as a basis for future targeted functional investigations.

However, this study presents some limitations. The post‐mortem human myotubes may not fully reflect the dynamic responses of muscle cells in living organisms [38]. Nevertheless, our experimental model relies on isolated CD56^+^ satellite cells that were expanded in culture and differentiated into myotubes prior to analysis, a process expected to attenuate transient post‐mortem transcriptional signatures. In line with this, all samples exhibited excellent RNA integrity, and previous studies have demonstrated that post‐mortem human satellite cells retain robust myogenic and functional competence able Stro [39, 40]. Despite these considerations, the absence of detailed clinical phenotyping associated with anonymized post‐mortem tissue procurement remains an inherent limitation. Therefore, our findings should be further validated able Svo using appropriate animal models and complemented by additional functional studies to confirm the biological relevance of the transcriptomic changes induced by UA and UB. In addition, although the paired analytical design (design = ~Patient + Treatment) accounts for donor‐specific baseline transcriptional differences, the limited sample size and unbalanced sex distribution (six men/three women; mean age ≈ 74 years, range 55–96 years) precluded formal age‐ or sex‐stratified analyses of UA‐ or UB‐induced responses. The primary objective of the present study was to estimate the average within‐donor treatment effect across individuals. While inter‐individual variability in response cannot be excluded, exploration of treatment‐by‐age or treatment‐by‐sex interactions would require larger and sex‐balanced cohorts and was beyond the scope of the current study.

In conclusion, this study provides a transcriptomic analysis of the molecular responses induced by UA and UB in human myotubes. Our results show that UA and UB elicit distinct and partially overlapping transcriptional responses, with UA preferentially modulating pathways related to mitochondrial function and inflammatory signalling, and UB affecting genes associated with lipid metabolism and muscle‐related processes. These findings, obtained in an able Stro model, offer mechanistic insights into how gut‐derived metabolites of pomegranate influence muscle cell biology and provide a rationale for future able Svo and clinical studies.

## Conflicts of Interest

The authors declare no conflicts of interest.

## Supporting information


**Table S1:** Primer sequences for qPCR.
**Table S2:** List of genes commonly up‐ and down‐regulated by UA and UB.
**Table S3:** Key biological functions and associated diseases in response to UA at 5 μM. Summary of the most significant biological functions and associated diseases, highlighting genes that are up‐regulated and down‐regulated in response to UA exposure.
**Table S4:** Key biological functions and associated diseases in response to UB at 5 μM. Summary of the most significant biological functions and associated diseases, highlighting genes that are up‐regulated and down‐regulated in response to UB exposure.
**Table S5:** Differentially expressed genes identified by DESeq2 in human myotubes treated with UA or UB compared with control conditions. The table reports Log2FoldChange, adjusted p‐value (padj), and gene annotations. Genes were identified using DESeq2 and subsequently used for pathway analysis.
**Figure S1:** Dynamics of protein expression during myogenesis. Desmin (A), MyoD1 (B), Pax7 (C) and MYHs (D). The dotted line represents the change of medium after 48 h of proliferation. Total RNA was isolated in cellular extract and Desmin, MyoD1, Pax7 and MYHs were analysed by Real Time PCR technology. Results were represented in mean with SEM (n = 7). * p < 0.05, ** p < 0.01 and *** p < 0.001.
**Figure S2:** Expression of myogenic markers through myogenesis highlighted by immunofluorescence. (A) Desmin is represented in red and DAPI is in blue. The arrow with square dots indicates a myoblast. The uninterrupted arrow indicates a myotube. (B) MyoD1 is represented in red and DAPI is in blue. The arrow with rectangular dots indicates a myoblast committed to return to a quiescent state. The arrow with square dots indicates a myoblast committed to differentiation. The uninterrupted arrow indicates a myotube. (C) Pax7 is represented in green and DAPI is in blue. The arrow with rectangular dots indicates a myoblast committed to return to a quiescent state. The arrow with square dots indicates a myoblast that is probably still in proliferation or in differentiation and not committed to quiescence. The uninterrupted arrow indicates a myotube. (D) Pax7 is represented in green. The arrow with rectangular dots indicates a myoblast committed to return to a quiescent state. The arrow with square dots indicates a myoblast that is probably still in proliferation or in differentiation and not committed to quiescence. The uninterrupted arrow indicates a myotube. (E) MYHs are represented in green and DAPI is in blue. The arrow with square dots indicates a myoblast. The uninterrupted arrow indicates a myotube. The antibody used target all isoforms of MYHs (20× magnification). The scale bars at the lower right represent 100 μm.
**Figure S3:** Distinct and Common Pathways influenced in the presence of UA or UB.
**Figure S4:** **Monitoring of myoblast differentiation dynamics using the Nanolive CX‐A microscope under control conditions and following treatment with UA and UB (5 μM)**. Representative fields are shown at baseline (T0) and after 6 h, 12 h, 18 h and 24 h of treatment. Nuclei were manually annotated and classified as isolated nuclei (pink) or nuclei adjacent to other nuclei within continuous cellular structures (green). Each image measures 3976 x 3976 pixels, with a pixel size of 0.2 μm, resulting in an 800 μm x 800 μm field of view.
**Figure S5:** **Quantification of isolated myoblasts during differentiation under control conditions and following treatment with UA or UB (5 μM)**. The number of isolated nuclei (mononucleated myoblasts) was manually counted at baseline (T0) and at 6, 12, 18, and 24 h after treatment initiation. Values were normalized to their respective T0 condition (set to 100%).

## Data Availability

The transcriptomic data analysed for this study are deposited in the Gene Expression Omnibus (GEO) repository under accession number GSE230047 and are publicly accessible at https://www.ncbi.nlm.nih.gov/geo/query/acc.cgi?acc=GSE230047.
