# Supplementary material for: Exploring the Impact of Urolithins A and B on Muscle Health: A Transcriptomic Analysis in Human Myotubes
Source: J Cachexia Sarcopenia Muscle. 2026 Sep 23;17(5):e70385. doi: 10.1002/jcsm.70385 (PMC13598681; doi:10.1002/jcsm.70385)
Supplement: Supplementary file 1 [file JCSM-17-e70385-s001.docx]

1 **Exploring the impact of Urolithins A and B on muscle health: A transcriptomic analysis in human**
2 **myotubes**

# 3

4 Yves Henrotin ^1,2^, Antoine Florin^1^, Christelle Sanchez^1^, Prescilia Centonze^1^, Tiago Pinto Coelho^3^, Sophie Bekisz^4^, Jean-Emile Dubuc^5^, Olivier Galand^6^, Cécile Lambert^1^

# 6

7

8 **Supplementary Methods**
9 ***RNA sequencing and differential gene expression analysis***
10 This analysis used one hundred nanograms of RNA from each culture condition. Libraries were
11 prepared with the Illumina Truseq stranded mRNA sample prep kit according to the manufacturer's
12 instructions.	The coding strand information was kept based on the poly(A) selection of mRNAs.
13 Poly(A) plus RNA was enriched using oligo(dT) beads, followed by fragmentation and reverse
14 transcription. Afterward, cDNA fragments 5' and 3' ends were prepared to ensure efficient ligation of "Y"
15 adapters containing a unique barcode and primer binding sites. Finally, ligated cDNAs were PCR-
16 amplified to be ready for cluster generation and sequencing. Sequencing was performed on Novaseq
17 (Illumina), paired end reads (150-10-10-150), NovaSeq S4 V1.5 300 cycles XP workflow, generating
18 around 20 M reads per sample. Denatured NGS library fragments were flowed across a flow cell and
19 hybridized on a lawn of complementary Illumina adapter oligos. Complementary fragments were
20 extended, amplified via bridge amplification PCR, and denatured, resulting in clusters of identical single-
21 stranded library fragments. Fragments were primed and sequenced utilizing reversible terminator
22 nucleotides. Base pairs were identified after laser excitation and fluorescence detection. Raw data were
23 demultiplexed into individual libraries. After filtering out reads mapping to rRNA, tRNA, mitochondrial
24 RNA, and other contaminants (e.g., adapters, etc.) using bowtie2, reads were aligned onto the human
25 reference genome (GRCh38 and Ensembl 106 annotation) and quantified with Star to give the Counts
26 file. Quality control of sequencing reads was assessed with FASTQC, and quality control was
27 assessed after mapping with Picard tools. Compilation of tool metrics was performed with MultiQC.
28 Differential expression analysis was made in R (version 4.4.0, [https://www.R-project.org/](https://www.r-project.org/)) using the
29 DESeq2 package (1.44.0) ^S1^, biomaRt package (version 2.60.0) ^S2^ and R code design = ~ Patient +
30 Treatment. Analysis was performed with treatment as a contrast: each treated vs control and the
31 combination vs each extract separately. Volcano plots and Venn diagrams were generated using ggplot2
32 (version 3.5.1) and nVennR (version 0.2.3) ^S3^. DEseq2 data were analyzed with the use of QIAGEN IPA
33 version 111725566 (QIAGEN Inc., https://digitalinsights.qiagen.com/IPA). The networks’ function and
34 disease analyses, and canonical pathway analyses were generated through QIAGEN IPA. A false
35 Discovery Rate (FDR) of 0.01 was used to assess the statistical significance. This stricter threshold was
36 chosen to minimize the rate of false positives. By applying a 0.01 threshold, we aimed to strike a balance
37 between detecting meaningful biological changes and controlling the overall false discovery rate,
38 aligning with best practices in the field of transcriptomics.

# 39

40

41 **Supplementary Tables**
42 **Supplementary Table S1. Primer sequences for qPCR**

| Gene | Primer sequence (5’→3’) |
| --- | --- |
| *S18* | F: CGGCTACCACATCCAAGGAA |
|  | R: GCTGGAATTACCGCGGCT |
| *NOTCH1* | F: GCACTGCGAGGTCAACA |
|  | R: GCACTTGGCACCATTCT |
| *MYMX* | F: CCAGCCAGAGACTGATTC |
|  | R: CAGGGGCAGGAGGTATT |
| *PANX1* | F: GGCTGCCTTTGTGGATT |
|  | R: GCTGCGAAACGCCAGAA |
| *FGF9* | F: GGACTCTACCTCGGGAT |
|  | R: TGAATTTCTGGTGCCGTT |
| *ICAM5* | F: CGGACTGGGACTGTTTGA |
|  | R: GTAGACCCTGGCCTCTGA |
| *STC2* | F: ATCCAGCACTGTTTGGT |
|  | R: CAAGGCGTCTTTGATGAA |
| *IL-17B* | F: AGAGGAACATCGAGGAGA |
|  | R: GCAGGTCCACGGGGATA |
| *PTGS1* | F: TTGACCGCTACCAGTGT |
|  | R: TCGGATGAAGGTGGCATT |

# 43

44

45	**Supplementary Table S2. List of genes commonly up- and down-regulated by UA and UB**

| **Gene**  **symbols** | **Common Down**  **(n=162)** | | | | | | | | **Common Up**  **(n=18)** |
| --- | --- | --- | --- | --- | --- | --- | --- | --- | --- |
|  | CAMKK1 | CARMIL1 | FAM83E | NECAB3 | SECTM1 | IL6R | RARG | MYBPC1 | ANLN |
|  | CD79B | KCNK2 | NAMPT | STAT5A | RNF157 | PLCD3 | CD7 | CACNA1H | MYLK |
|  | SCN4A | NKAIN1 | RARRES2 | FBXL16 | PRDM15 | ADCY9 | GLIS1 | MAML3 | ATP2B1 |
|  | JARID2 | YPEL3 | P2RX1 | FGL2 | SLC44A3 | SNED1 | CSRP2 | PDGFA | CDC6 |
|  | LTF | CCDC80 | SH3D19 | GNAI1 | IGS1F1 | RFTN2 | TCIM | PLXNB3 | SHD |
|  | PLEKH01 | PTGS1 | ST3GAL4 | CDKN1C | SLC43A1 | IGFN1 | RPRM | RBM20 | RND2 |
|  | HOXC8 | PALMD | MANSC1 | NPAS1 | KIAA1755 | PTPN13 | FAM20C | COL15A1 | OGFRL1 |
|  | BEST2 | CECR2 | EXOC2 | RFTN1 | ADRA2A | TIPARP | EXOC3L1 | ITSN1 | PDLIM4 |
|  | LTBP1 | GRK3 | STC2 | KANK4 | ITPR1 | LRRC2 | TMTC2 | GPX3 | ANXA1 |
|  | ELN | PIK3IP1 | PODXL2 | PTGFRN | ANK3 | TPRA1 | SLC9A9 | LINC00511 | GGH |
|  | DKK3 | DMC1 | REEP6 | CD101 | DPYSL4 | YEATS2 | MAFA | PSG1 | ABHD2 |
|  | COL23A1 | TRIM9 | PLCD4 | ARMC9 | PID1 | ARHGEF3 | TMEM119 | SELENOP | RNF217 |
|  | AHRR | SLCO4A1 | NFE2L2 | IER3 | ABI3BP | ZNF704 | MAFF | LINC02995 | CDKN2B |
|  | SLC12A2 | NKAIN4 | NEK6 | FGFBP2 | PDE1C | PKNOX2 | LRRC14B | SMIM3 | KRT80 |
|  | CALCRL | ST8SIA5 | PCDHB10 | FXYD6 | APCDD1 | TCP11L2 | MYADML2 | RAB7B | FILIP1L |
|  | SDK2 | SGCG | TGFBI | ITGA11 | ELMO1 | TMEM100 | FAM43A |  | DIPK2A |
|  | RPS6KA2 | OCA2 | TXB2 | CYP1B1 | ART3 | PLEKHA7 | TNFAIP2 |  | MYBL1 |
|  | TP63 | BMF | LHX4 | HECW2 | ACAN | MIDN | UBALD2 |  | CYRIA |
|  | NTN4 | STMN2 | FAM124B | SHISAL1 | DPYSL5 | ZNF608 | PRKAR1B |  |  |
|  | CAPN6 | OLFM2 | CHD6 | LUM | CLSTN2 | NPAS2 | SLC38A3 |  |  |
|  | GPR137B | TGFB1 | BMP4 | MYLK3 | GDPD5 | UGT2B7 | FZD9 |  |  |

# 46

**Supplementary Table S3. Key biological functions and associated diseases in response to UA at 5 µM. Summary of the most significant biological functions and associated diseases, highlighting genes that are up-regulated and down-regulated in response to UA exposure.**

**Supplementary Table S4. Key biological functions and associated diseases in response to UB at 5 µM. Summary of the most significant biological functions and associated diseases, highlighting genes that are up-regulated and down-regulated in response to UB exposure.**

**Supplementary Table S5. Differentially expressed genes identified by DESeq2 in human myotubes treated with UA or UB compared with control conditions. The table reports Log2FoldChange, adjusted *p*-value (padj), and gene annotations. Genes were identified using DESeq2 and subsequently used for pathway analysis.**

47

64 **Supplementary Figures**

**Figure S1. Dynamics of protein expression during myogenesis. Desmin (A), MyoD1 (B), Pax7 (C) and MYHs (D). The dotted line represents the change of medium after 48 hours of proliferation. Total RNA was isolated in cellular extract and Desmin, MyoD1, Pax7 and MYHs were analysed by Real Time PCR technology. Results were represented in mean with SEM (n=7). * *P*< 0.05, ** *P*< 0.01 and *** *P*< 0.001.**

**Figure S2: Expression of myogenic markers through myogenesis highlighted by immunofluorescence. (A) Desmin is represented in red and DAPI is in blue. The arrow with square dots indicates a myoblast. The uninterrupted arrow indicates a myotube. (B) MyoD1 is represented in red and DAPI is in blue. The arrow with rectangular dots indicates a myoblast committed to return to a quiescent state. The arrow with square dots indicates a myoblast committed to differentiation. The uninterrupted arrow indicates a myotube. (C) Pax7 is represented in green and DAPI is in blue. The arrow with rectangular dots indicates a myoblast committed to return to a quiescent state. The arrow with square dots indicates a myoblast that is probably still in proliferation or in differentiation and not committed to quiescence. The uninterrupted arrow indicates a myotube. (D) Pax7 is represented in green. The arrow with rectangular dots indicates a myoblast committed to return to a quiescent state. The arrow with square dots indicates a myoblast that is probably still in proliferation or in differentiation and not committed to quiescence. The uninterrupted arrow indicates a myotube. (E) MYHs are represented in green and DAPI is in blue. The arrow with square dots indicates a myoblast. The uninterrupted arrow indicates a myotube. The antibody used target all isoforms of MYHs (20x magnification). The scale bars at the lower right represent 100µm.**

66 **Figure S3. Distinct and Common Pathways influenced in the presence of UA or UB**
67 See attached file.

# 50

**Figure S4. Monitoring of myoblast differentiation dynamics using the Nanolive CX-A microscope under control conditions and following treatment with UA and UB (5 µM).** Representative fields are shown at baseline (T0) and after 6 h, 12 h, 18 h and 24 h of treatment. Nuclei were manually annotated and classified as isolated nuclei (pink) or nuclei adjacent to other nuclei within continuous cellular structures (green). Each image measures 3976 x 3976 pixels, with a pixel size of 0.2 µm, resulting in an 800 µm x 800 µm field of view.

**Figure S5. Quantification of isolated myoblasts during differentiation under control conditions and following treatment with UA or UB (5 µM).** The number of isolated nuclei (mononucleated myoblasts) was manually counted at baseline (T0) and at 6, 12, 18, and 24 h after treatment initiation. Values were normalized to their respective T0 condition (set to 100%).

68 **Supplementary References**
69 S1. Love MI, Huber W, Anders S. Moderated estimation of fold change and dispersion for RNA-seq data with DESeq2. *Genome Biol* 2014;15.
70 S2. Durinck S, Spellman PT, Birney E, Huber W. Mapping identifiers for the integration of genomic datasets with the R/Bioconductor package biomaRt. Nat Protoc 2009;4:1184–1191.
71 S3. Pérez-Silva JG, Araujo-Voces M, Quesada V. nVenn: generalized, quasi-proportional Venn and Euler diagrams. Bioinformatics 2018;34:2322–2324.
72 S4. Hochberg Y. A Sharper Bonferroni Procedure for Multiple Tests of Significance. *Biometrika* 1988;75:800.
73 S5. Hommel G. A stagewise rejective multiple test procedure based on a modified bonferroni test. *Biometrika* 1988;75:383–386.
74 S6. Benjamini Y, Hochberg Y. Controlling the False Discovery Rate: A Practical and Powerful Approach to Multiple Testing. Journal of the Royal Statistical Society: Series B (Methodological) 1995;57:289–300.
75 S7. Benjamini Y, Yekutieli D. The control of the false discovery rate in multiple testing under dependency. *Ann Stat* 2001;29:1165–1188.
